# Effect of LongZhang Gargle on Biofilm Formation and Acidogenicity of* Streptococcus mutans In Vitro*

**DOI:** 10.1155/2016/5829823

**Published:** 2016-05-25

**Authors:** Yutao Yang, Shiyu Liu, Yuanli He, Zhu Chen, Mingyun Li

**Affiliations:** ^1^State Key Laboratory of Oral Diseases, Sichuan University, Chengdu 610041, China; ^2^Department of Operative Dentistry and Endodontics, West China Hospital of Stomatology, Sichuan University, Chengdu 610041, China; ^3^Wuhan First Hospital of Stomatology, Jianghan University, Wuhan 430065, China; ^4^Guiyang Hospital of Stomatology, Guiyang 550002, China

## Abstract

*Streptococcus mutans*, with the ability of high-rate acid production and strong biofilm formation, is considered the predominant bacterial species in the pathogenesis of human dental caries. Natural products which may be bioactive against* S. mutans* have become a hot spot to researches to control dental caries. LongZhang Gargle, completely made from Chinese herbs, was investigated for its effects on acid production and biofilm formation by* S. mutans* in this study. The results showed an antimicrobial activity of LongZhang Gargle against* S. mutans* planktonic growth at the minimum inhibitory concentration (MIC) of 16% and minimum bactericidal concentration (MBC) of 32%. Acid production was significantly inhibited at sub-MIC concentrations. Biofilm formation was also significantly disrupted, and 8% was the minimum concentration that resulted in at least 50% inhibition of biofilm formation (MBIC_50_). A scanning electron microscopy (SEM) showed an effective disruption of LongZhang Gargle on* S. mutans* biofilm integrity. In addition, a confocal laser scanning microscopy (CLSM) suggested that the extracellular polysaccharides (EPS) synthesis could be inhibited by LongZhang Gargle at a relatively low concentration. These findings suggest that LongZhang Gargle may be a promising natural anticariogenic agent in that it suppresses planktonic growth, acid production, and biofilm formation against* S. mutans*.

## 1. Introduction

Dental caries is a chronic, progressive, and infectious disease which happens to the hard tissue of the teeth [1]. It is one of the most prevalent chronic human infectious diseases worldwide [2, 3]. The progress begins with extensive destruction of the enamel and dentin, followed by cavitation, inflammation of the pulp, and periapical tissue, even ending with tooth loss [4]. Unlike most infectious diseases exhibiting classic virulence factors such as endotoxin (lipopolysaccharide, LPS), the etiologic factors of dental caries are initiated with the bacterial adherence to the tooth surface and dental plague biofilm formation. After that, cariogenic bacteria within dental plague biofilm produce acid by metabolizing carbohydrates ingested from the host, leading to demineralization of enamel surfaces and the development of caries [5, 6].

*Streptococcus mutans *(*S. mutans*) has been identified as the major pathogen of human dental caries [5, 7], although additional microorganisms may be involved [8, 9]. Reasons are as follows:* S. mutans* can easily adhere to enamel surfaces, it can synthesize extracellular polysaccharides (EPS) in the presence of carbohydrate, which mediates the irreversible adhesive interaction between bacterial cells and forms a high-cell-density biofilm [10], and it exhibits a high-rate acid production and is highly aciduric, which allows it to survive and continue to produce acids in low pH microenvironments [11, 12].

Many products are being developed for caries control. Among them, the most effective and widely used cariostatic agent is fluoride. It prevents dental caries by inhibiting demineralization and enhancing remineralization on the enamel surface. However, side effects such as fluorosis limited its use for public health [13, 14]. The antimicrobials including chlorhexidine and antibiotics have also been used to prevent dental caries. However, they may cause tooth and tongue discoloration, host cytotoxicity, pathogen drug resistance, and disturbance of oral flora [15–17]. Therefore, alternative agents for caries control with minimal adverse effects are promising.

In recent years, natural products which may be bioactive against* S. mutans* have been of great interest to the researchers in the field of caries control, mainly due to their few side effects. Currently, many cariostatic natural products have been identified, typical natural products such as ginkgoneolic acid [4], epigallocatechin gallate [18, 19], and cranberry polyphenols [20]. LongZhang Gargle, completely made from Chinese folk herbs, was created and used by Miao people, who are the ethnic minorities mainly living in Southwest China. Industrially, it has been manufactured by Guiyang Xintian Pharmaceutical Company, Guizhou, China, for nearly 20 years. The components of the Gargle include the root and leaves of* Toddalia asiatica *(L.) Lam., Cortex Lycii, and* Cimicifuga foetida*.* Toddalia asiatica* (L.) Lam. (*T. asiatica*), as the main component of LongZhang Gargle, belongs to the familyRutaceae and is a woody liana that grows in tropical and subtropical areas of the world. It is widely recognized as a medicinal plant in China, India, Japan, and Africa. All parts of the plant are believed to have medicinal properties, and its root and leaves have been processed to treat many diseases, such as rheumatism, influenza, malaria, indigestion, stomachache, and toothache [21, 22]. Currently, the pharmacological effects of* T. asiatica* considered mainly include antipyretic, antinociceptive, anti-inflammatory, and antimicroorganism. For example, a study showed the antinociceptive and anti-inflammatory effects of its root extract on Swiss albino mice [23]. Some studies also demonstrated the antibacterial and antifungal activities of* T. asiatica *extracts by measuring the planktonic growth of various kinds of microorganism, such as* Staphylococcus aureus*,* Staphylococcus epidermidis*,* Escherichia coli*,* Pseudomonas aeruginosa*,* Klebsiella pneumoniae*, and* Candida albicans *[22, 24–26]. At present, LongZhang Gargle is mainly applied for treating gingivitis, periodontitis, and oral ulcer on clinic [27, 28]. It can also significantly reduce bacteria in root canals and act as an effective root canal disinfectant [29]. Although there are many clinical researches confirming the value of LongZhang Gargle for curing oral diseases, studies on pharmacological mechanism of it are still scarce. Moreover, the studies of* T. asiatica* on microorganism have all been performed in planktonic cultures and the existing researches completely ignore the effect of* T. asiatica *on oral biofilms. In addition, cariogenic bacteria such as* S. mutans* have never been studied either.

Considering the antimicrobial effect of* T. asiatica* and LongZhang Gargle as previous studies showed, we suppose that the gargle can inhibit the growth of* S. mutans* in planktonic culture. Our research also aims to explore the effects of LongZhang Gargle on virulence factors of* S. mutans* including biofilm formation and acid production. The study contributes to the finding of a new natural medication treatment of dental caries without any significant side effects, as well as the finding of the multiple uses of LongZhang Gargle, so as to promote LongZhang Gargle at a wider clinical application.

## 2. Materials and Methods

### 2.1. Chemicals, Bacterial Strains, and Growth Conditions

The agent of LongZhang Gargle was provided by Guiyang Xintian Pharmaceutical Co. Ltd., Guizhou, China.* S. mutans* UA159 was provided by State Key Laboratory of Oral Diseases, Sichuan University, Chengdu, China. Brain-heart infusion (BHI) broth was used for investigating the minimum inhibitory concentration (MIC), minimum bactericidal concentration (MBC), and acidogenicity. For the biofilm formation, 1% (wt/vol) sucrose was added to BHI (BHIS). Alexa Fluor 647-labeled dextran conjugate, as a red fluorescent stain for EPS, was bought from Invitrogen, Carlsbad, CA, USA. SYTO 9, as a green fluorescent nucleic acid stain, was bought from Molecular Probes, Eugene, OR, USA. The bacterium was grown at 37°C under anaerobic conditions (5% CO_2_).

### 2.2. Minimum Inhibitory Concentration (MIC) and Minimum Bactericidal Concentration (MBC)

The MIC and MBC of LongZhang Gargle against* S. mutans *were determined by liquid medium double dilution method modified from that of Lombardo Bedran et al. [30]. The wells of a 96-well tissue culture plate, each containing 190 *μ*L of serially diluted LongZhang Gargle (1%, 2%, 4%, 8%, 16%, 32%, and 64%) in BHI culture media, were inoculated with 10 *μ*L of an overnight culture of* S. mutans* diluted in fresh BHI broth to obtain an OD_600_ of 0.2 (about 10^8^ CFU/mL). Each concentration contained three parallel samples and BHI broth without gargle was used as a control. The MIC was the lowest concentration of the gargle that no bacteria were grown in the broth. To determine the MBC, the 200 *μ*L cultures with 10 *μ*L of* S. mutans* and 190 *μ*L of BHI at a drug concentration above MIC were aspirated into BHI agar plates and incubated at 37°C for 24 h. The MBC was the lowest concentration of the gargle that no bacteria could be observed on the agar plate.

### 2.3. Effect of LongZhang Gargle on Biofilm Formation

The effect of LongZhang Gargle on* S. mutans* biofilm formation was measured by the method modified from that of Li et al. [31] and Assaf et al. [32]. Overnight-grown* S. mutans* was diluted in BHI broth to obtain an OD_600_ of 0.2 (about 10^8^ CFU/mL). The wells of a sterile 96-well tissue culture plate, each contained 10 *μ*L of such cell suspension and 190 *μ*L of BHIS broth with different LongZhang Gargle concentrations below the MIC (1%, 2%, 4%, and 8%), were incubated for a biofilm formation. Each concentration contained three parallel samples and BHIS broth without gargle was used as a control. After 24 h, supernatant from each well was aspirated, and the biofilm in each well was fixed with methanol for 15 min, stained with crystal violet (0.5%) for 30 min, and washed three times with distilled deionized water to remove the unbound crystal violet. After that, 200 *μ*L of 100% ethanol was added to each well to dissolve the crystal violet on the biofilm. The plate was rocked at room temperature for 20 min, and the absorbance was read at 600 nm by a spectrophotometer.

### 2.4. Effect of LongZhang Gargle on Biofilm Morphology

The structure of* S. mutans* biofilms formed in the presence of LongZhang Gargle was observed by scanning electron microscopy modified from that of Bitoun et al. [33] and Jongsma et al. [34]. Briefly, 100 *μ*L of overnight-grown* S. mutans* suspension diluted in fresh BHI at an initial OD_600_ of 0.2 and 1900 *μ*L BHIS broth with different concentrations of LongZhang Gargle below the MIC (1%, 2%, 4%, and 8%) was added to the wells of a 24-well tissue culture plate. Each concentration contained three parallel samples and BHIS broth without gargle was used as a control. Glass coverslips (5 mm in diameter) were prefixed in each well. After incubation for 24 h, the biofilm-coated glass coverslips were immersed in 2.5% glutaraldehyde at 4°C overnight, washed three times with distilled deionized water, dehydrated using ascending graded serious of ethanol (30%, 50%, 70%, 80%, 85%, 90%, 95%, and 100%), and coated with gold. The samples were then examined by SEM.

### 2.5. Effect of LongZhang Gargle on Extracellular Polysaccharide (EPS) Synthesis and Bacterial Viability

The* S. mutans* biofilms were observed for the volume of their major components (EPS and bacteria) by confocal laser scanning microscopy modified from Xiao and Koo [35] and Qiu et al. [36] as described previously. 100 *μ*L overnight-grown* S. mutans* diluted in fresh BHI at an initial OD_600_ of 0.2 was added to the wells of a 24-well tissue culture plate and grown in 1900 *μ*L BHIS with 0, 1%, 2%, and 4% of LongZhang Gargle, respectively. Sterile slides were prefixed in each well. 1 *μ*L of 2.5 *μ*M Alexa Fluor 647-labeled dextran conjugate was added to each well during the formation of biofilms. After incubation for 24 h, the biofilms were washed three times with PBS and stained with 50 *μ*L of 2.5 *μ*M SYTO 9 green fluorescent nucleic acid stain at 4°C for 30 min. Then the samples were examined by a confocal laser scanning microscope (CLSM) under 63x oil immersion objectives (Figure 3). Two samples and three fields per sample were observed for each group.

### 2.6. Effect of LongZhang Gargle on Acidogenicity

The effect of LongZhang Gargle on acidogenicity of* S. mutans *was examined by a standard pH drop. We modified the measure from that of Xu et al. [19]. Bacteria were harvested at mid-logarithmic phase, washed with phosophate-buffered saline (PBS), and grown in potassium phosphate buffer with glucose (contained 0.5 mM K_2_HPO_4_, 0.5 mM KH_2_PO_4_, 37.5 mM KCl, 1.25 mM MgCl_2_, and 1% wt/vol glucose, initial pH was 6.50). The initial optical density (OD) of the mixture at 600 nm was 0.5. LongZhang Gargle was then added to reach different concentrations below the MIC (1%, 2%, 4%, and 8%), as well as a nontreated control. The decrease in pH, as a result of glycolytic activity of* S. mutans* UA159, was monitored at 15 min intervals over a period of 120 min.

### 2.7. Statistical Analysis

All the experiments were repeated at least three times. Differences between the experiment group and the untreated control group were statistically analyzed by SPSS (version 20.0 for Windows). One-way ANOVA and post hoc Tukey's multiple-comparison test were applied for the comparison of multiple means. The chosen level of significance was set at *P* < 0.05.

## 3. Results

### 3.1. LongZhang Gargle Exhibits Antimicrobial Activity against* S. mutans*

We determined the antimicrobial effect of LongZhang Gargle by measuring MIC and MBC against* S. mutans*. The results showed that LongZhang Gargle inhibited the growth of planktonic* S. mutans* UA159 at a MIC of 16% and MBC of 32%.

### 3.2. *S. mutans *Biofilm Is Susceptible to LongZhang Gargle

**Figure 1:**
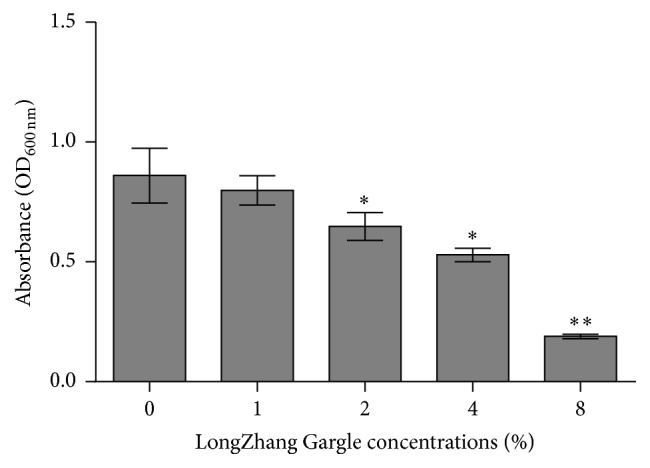
Effect of LongZhang Gargle on biofilm formation.* S. mutans* was incubated in BHIS broth for 24 h with LongZhang Gargle at concentrations of 0, 1% (1/16 MIC), 2% (1/8 MIC), 4% (1/4 MIC), and 8% (1/2 MIC). ^*∗*^ *P* < 0.05, ^*∗∗*^ *P* < 0.01.

The effect of LongZhang Gargle on biofilm formation by* S. mutans* was determined by measuring the absorbance at 600 nm in crystal violet assay. As is shown in Figure 1, the results were 0.860, 0.798, 0.648, 0.529, and 0.189 relative light units at the concentrations of 0, 1% (1/16 MIC), 2% (1/8 MIC), 4% (1/4 MIC), and 8% (1/2 MIC), respectively. Statistical analysis indicated that LongZhang Gargle significantly inhibited* S. mutans *biofilm formation at 2% (*P* < 0.05), 4% (*P* < 0.05), and 8% (*P* < 0.01). No significant difference was found between control (0%) and 1%. Furthermore, differences are significant between 1% and 2% (*P* < 0.05), 2% and 4% (*P* < 0.05), and 4% and 8% (*P* < 0.001). This demonstrated that the inhibitory effect was dosage dependent. The percentage of inhibition was calculated using the following equation: (1 − *A*_600_ of the test group/*A*_600_ of blank control) × 100%. And the inhibition percentages are 38.5% for concentration of 4% and 78.0% for 8%. So 8% was considered as MBIC_50_ which meant the minimum concentration that resulted in at least 50% inhibition of biofilm formation compared with that in control.

**Figure 2:**
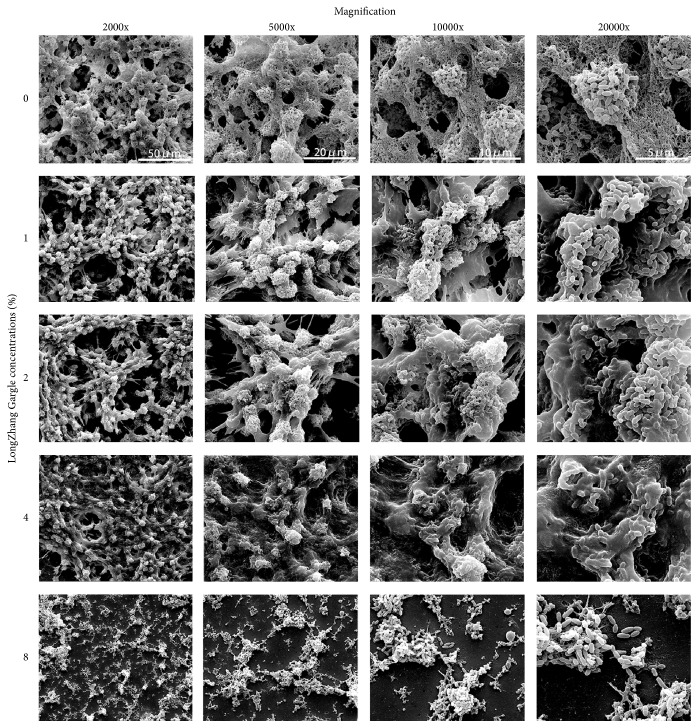
Effect of LongZhang Gargle on biofilm morphology.* S. mutans* was incubated for 24 h in BHIS broth, with LongZhang Gargle at concentrations of 2%, 4%, and 8%. The control group without drug was also conducted. Magnification was 2000x, 5000x, 10000x, and 20000x, respectively, for each concentration.

**Figure 3:**
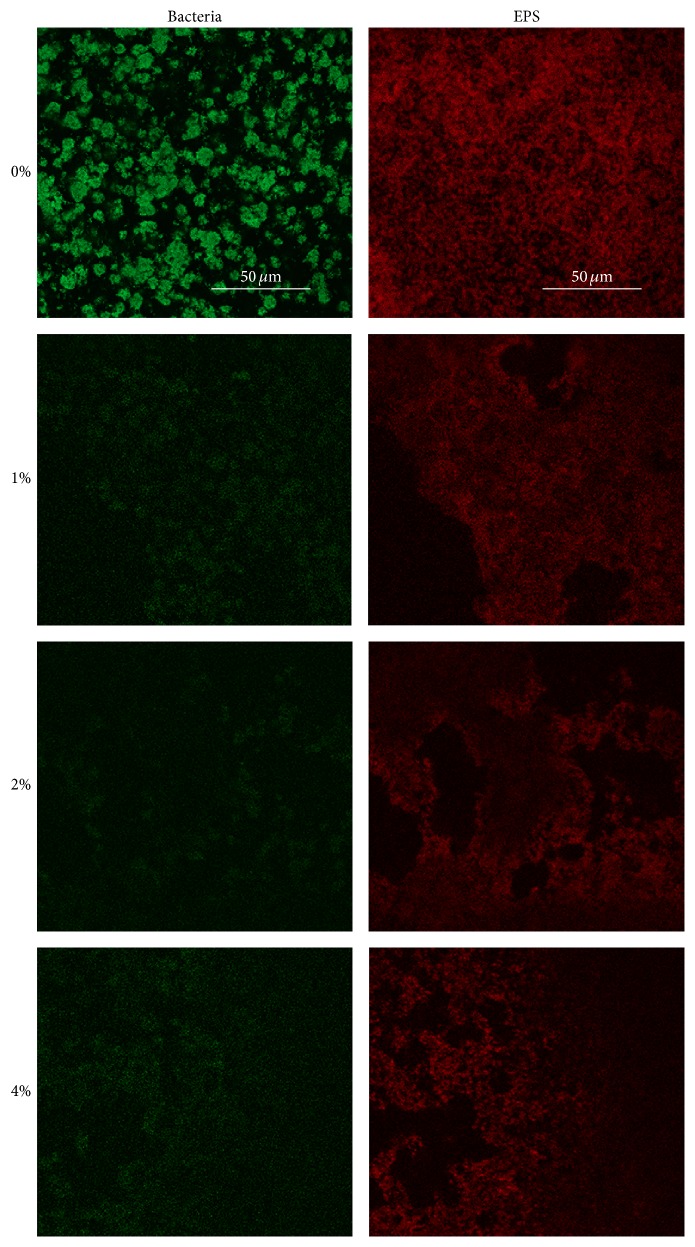
Effect of LongZhang Gargle on Extracellular Polysaccharide (EPS) Synthesis of* Streptococcus mutans*. The cells were treated with LongZhang Gargle at concentrations of 0, 1% (1/16 MIC), 2% (1/8 MIC), and 4% (1/4 MIC) for 24 h. EPS was labeled red (Alexa Fluor 647) and bacterial cells were labeled green (SYTO 9) under a confocal laser scanning microscope. Magnification was 63x for oil immersion objective.

The morphology alteration of* S. mutans* biofilm was observed by SEM. As is shown in Figure 2, thick and relatively thick biofilm was formed in control (without the gargle). However, biofilm integrity was disrupted when treated with different concentrations of LongZhang Gargle. With a concentration of 8% (MBIC_50_), only scattered bacteria could be seen.

**Figure 4:**
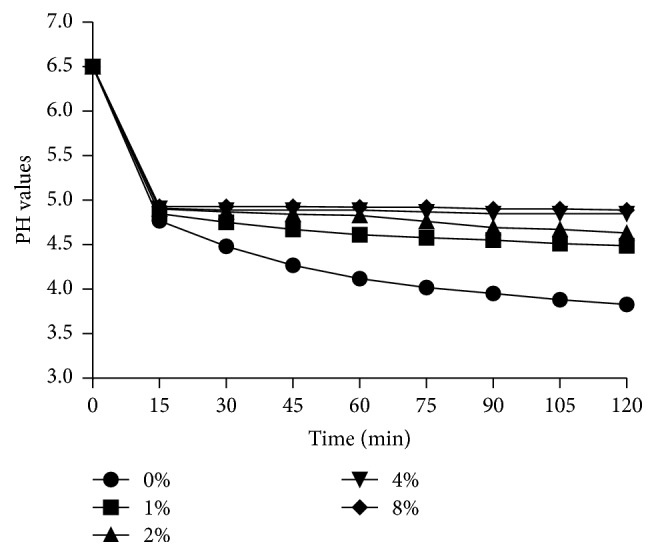
Effect of LongZhang Gargle on the acid production of* S. mutans*.* S. mutans* was incubated in BHI broth with LongZhang Gargle at concentrations of 0, 1% (1/16 MIC), 2% (1/8 MIC), 4% (1/4 MIC), and 8% (1/2 MIC). Glycolytic acid production was determined by monitoring the pH drop in glucose solution (1% wt/vol) at 15 min intervals over a period of 120 min.

The major components of* S. mutans* biofilms are EPS and bacteria. However, crystal violet assay and scanning electron microscopy cannot accurately differentiate one from the other. Therefore, we conducted the CLSM study to investigate how these two components were affected by LongZhang Gargle. As is shown in Figure 4, the control group (without the gargle) witnessed a high density of both EPS and bacteria cells. The intensity of fluorescence, red for EPS and green for bacteria cells, was both significantly decreased when LongZhang Gargle added. A higher concentration seemed to contribute a sharper decrease for the volume of both EPS and bacteria. To conclude, the results of crystal violet assay, SEM, and CLSM all suggest that LongZhang Gargle could inhibit the formation of* S. mutans* biofilm.

### 3.3. LongZhang Gargle Reduces Acid Production of* S. mutans*

We determined the effect of LongZhang Gargle on acidogenicity of* S. mutans* by monitoring the glycolytic pH drop in planktonic culture. As is shown in Figure 4, the acid production of* S. mutans* UA159 was inhibited by LongZhang Gargle at sub-MIC levels. Higher concentration (1/2 MIC, 8%) contributed a more obvious inhibitory effect on pH drop than other concentrations. Also, the initial 30 min showed the highest pH drop.

## 4. Discussion

Natural products are a rich source for discovery of new antimicrobial agents [37]. Yet we know very little about an antimicrobial effect of LongZhang Gargle, a traditional Chinese herbal compound. In this study, we found that LongZhang Gargle showed an antimicrobial effect on* S. mutans *in planktonic culture. Diluted LongZhang Gargle at concentration of 16% could effectively and completely inhibit the growth of* S. mutans* and at the concentration of 32% showed an obvious bactericidal activity. Considering that high concentration of the drug may cause side effects, the MIC and MBC may help to determine the appropriate concentration for applying it. However, the bacteria in human mouth exist in biofilms rather than planktonic form, and other oral bacterial species in biofilms probably also contribute to oral diseases. Therefore, we conducted the crystal violet assay, SEM and CLSM, to explore the effects of LongZhang Gargle on* S. mutans* biofilm formation as described above. For further study, other common cariogenic bacteria and biofilms formation could also be similarly investigated.

Dental caries is caused by oral microorganisms through metabolizing carbohydrate, producing acid, and demineralizing the tooth surface. Thus, the reduction of acid production is a logical approach to prevent dental caries. However, previous studies completely ignore the effects of LongZhang Gargle on acid production of oral bacteria. In our research,* S. mutans* was examined at mid-logarithmic phase to guarantee a best state of acidogenicity. The results showed that concentration of 1% (1/16 MIC) of LongZhang Gargle could inhibit acid production of* S. mutans*. This finding suggested that acidogenicity of* S. mutans* in biofilms could probably also be inhibited. Therefore, we may conduct further experiment to measure the influence of LongZhang Gargle on the change of pH of the* S. mutans* biofilm model. Moreover, complicated dental plaque naturally formed will also be the research object to be tested. Furthermore, since the acidogenicity inhibition concentration was very low (1/16 MIC), we hypothesize that the mechanism of the suppression may be attributed to the inhibition of enzymes that related to acid production, rather than bactericidal activity of the drug.

*S. mutans* is a bacterium primarily adhering to dental surface in dental plaque in the mouth. Biofilms provide protection against the action of antibiotics and supply a barrier to prevent or reduce the penetration of antimicrobial agents through the matrix [38] and are also considered as the initiating factor for dental diseases [39]. Therefore, the reduction of stable biofilm formation is an effective way to prevent dental caries. To the best of our knowledge, the effect of LongZhang Gargle on the* S. mutans* biofilm has not yet been documented. In this study, we identified that the biofilm of* S. mutans* was susceptible to LongZhang Gargle. In crystal violet assay, we found that biofilm formation was significantly suppressed at the concentration of 2% LongZhang Gargle, and this effect was dosage dependent. Moreover, we also found that the MBIC_50_ (8%) was lower than MIC (16%), and this result indicated that the gargle was more effective to inhibit aggregation of cells and reduce biofilm formation, compared with its bacteriostatic activity. In addition, under scanning electron microscopy, we observed that the biofilm was relatively thick and homogeneous without the gargle compared with those samples treated with gargle at different concentrations. As the concentration of the gargle increases, the biofilm integrity and structure is gradually being disrupted. Moreover, this disruption to biofilm integrity was also dosage dependent. At a concentration of 8%,* S. mutans* biofilm became very sparse and could not cover the surface of the slips, and the bacteria cluster became much smaller compared with the control group. Furthermore, for the confocal laser scanning microscopy, we found that the volume of EPS and bacterial cells became much less when adding the gargle, even though the concentration was relatively low. As we described above, the synthesis of EPS is a vital process for dental caries as it mediates irreversible adherence between bacteria and tooth surface, as well as aggregation between bacterial cells. Therefore, LongZhang Gargle can reduce the adherence and aggregation of the bacteria, followed by the inhibition of local pH drop on tooth enamel, which leads to dental caries.

Recently, anticariogenic-active natural products are gradually being studied. It has become a hot spot for researchers to explore traditional Chinese herbs that can prevent dental caries [40, 41]. LongZhang Gargle, completely made from traditional Chinese herbs, has been widely used since being manufactured 20 years ago, mainly applied for treating gingivitis, periodontal disease, and oral ulcer. Our study revealed the prospect of LongZhang Gargle as an anticariogenic treatment* in vitro*. Therefore, we suppose it can logically reduce caries incidence rate for susceptible population. If this speculation is correct, LongZhang Gargle will be an integrated drug to prevent and cure the common oral diseases, including dental caries, gingivitis, periodontal disease, and oral ulcer. To confirm our speculation, more clinical studies of LongZhang Gargle need to be conducted.

## 5. Conclusion

This study has identified that LongZhang Gargle owns an antimicrobial effect against* S. mutans*, and it is the first to identify the inhibitory effects on acid production and biofilm formation. This indicates that LongZhang Gargle might act as a promising way to control dental caries. However, further and more studies should be considered in future, for example, the effects on other cariogenic bacteria, such as* Actinomyces viscosus*, and the clinical study on dosage, efficacy, and side effects of LongZhang Gargle.

## References

[1] Selwitz R. H., Ismail A. I., Pitts N. B. (2007). Dental caries. *The Lancet*.

[2] Petersen P. E. (2003). The world oral health report 2003: continuous improvement of oral health in the 21st century—the approach of the WHO global oral health programme. *Community Dentistry and Oral Epidemiology*.

[3] Dye B. A., Tan S., Smith V. (2007). Trends in oral health status: United States, 1988–1994 and 1999–2004. *Vital and Health Statistics*.

[4] He J. Z., Wang S. D., Wu T. X., Cao Y. P., Xu X., Zhou X. D. (2013). Effects of ginkgoneolic acid on the growth, acidogenicity, adherence, and biofilm of *Streptococcus mutans* in vitro. *Folia Microbiologica*.

[5] Bowen W. H., Koo H. (2011). Biology of *Streptococcus mutans*-derived glucosyltransferases: role in extracellular matrix formation of cariogenic biofilms. *Caries Research*.

[6] Ma R., Sun M., Wang S. (2013). Effect of high-fructose corn syrup on the acidogenicity, adherence and biofilm formation of *Streptococcus mutans*. *Australian Dental Journal*.

[7] Smith E. G., Spatafora G. A. (2012). Gene regulation in *S. mutans*: complex control in a complex environment. *Journal of Dental Research*.

[8] Takahashi N., Nyvad B. (2008). Caries ecology revisited: microbial dynamics and the caries process. *Caries Research*.

[9] Tanzer J. M., Livingston J., Thompson A. M. (2001). The microbiology of primary dental caries in humans. *Journal of Dental Education*.

[10] Hamada S., Koga T., Ooshima T. (1984). Virulence factors of *Streptococcus mutans* and dental caries prevention. *Journal of Dental Research*.

[11] Banas J. A. (2004). Virulence properties of *Streptococcus mutans*. *Frontiers in Bioscience*.

[12] Matsui R., Cvitkovitch D. (2010). Acid tolerance mechanisms utilized by *Streptococcus mutans*. *Future Microbiology*.

[13] Featherstone J. D. B. (1999). Prevention and reversal of dental caries: role of low level fluoride. *Community Dentistry & Oral Epidemiology*.

[14] ten Cate J. M. B. (2009). The need for antibacterial approaches to improve caries control. *Advances in Dental Research*.

[15] Da Silva Pierro V. S., Barcelos R., Maia L. C., Da Silva A. N. (2004). Pediatricians' perception about the use of antibiotics and dental caries—a preliminary study. *Journal of Public Health Dentistry*.

[16] Arweiler N. B., Boehnke N., Sculean A., Hellwig E., Auschill T. M. (2006). Differences in efficacy of two commercial 0.2% chlorhexidine mouthrinse solutions: a 4-day plaque re-growth study. *Journal of Clinical Periodontology*.

[17] Scheie A. A., Petersen F. C., Fejerskov O., Kidd E. (2008). Antimicrobials in caries control. *Dental Caries, the Disease and Its Clinical Management*.

[18] Koyama Y., Kuriyama S., Aida J. (2010). Association between green tea consumption and tooth loss: cross-sectional results from the Ohsaki Cohort 2006 Study. *Preventive Medicine*.

[19] Xu X., Zhou X. D., Wu C. D. (2011). The tea catechin epigallocatechin gallate suppresses cariogenic vrrulence factors of *Streptococcus mutans*. *American Society for Microbiology*.

[20] Duarte S., Gregoire S., Singh A. P. (2006). Inhibitory effects of cranberry polyphenols on formation and acidogenicity of Streptococcus mutans biofilms. *FEMS Microbiology Letters*.

[21] Watanabe A., Kato T., Ito Y. (2014). Aculeatin, a coumarin derived from *Toddalia asiatica* (L.) Lam., enhances differentiation and lipolysis of 3T3-L1 adipocytes. *Biochemical and Biophysical Research Communications*.

[22] Duraipandiyan V., Ayyanar M., Ignacimuthu S. (2006). Antimicrobial activity of some ethnomedicinal plants used by Paliyar tribe from Tamil Nadu, India. *BMC Complementary and Alternative Medicine*.

[23] Kariuki H. N., Kanui T. I., Yenesew A., Patel N., Mbugua P. M. (2013). Antinocieptive and anti-inflammatory effects of *Toddalia asiatica* (L) Lam. (Rutaceae) root extract in Swiss albino mice. *The Pan African Medical Journal*.

[24] Hu J., Shi X., Chen J. (2014). Alkaloids from Toddalia asiatica and their cytotoxic, antimicrobial and antifungal activities. *Food Chemistry*.

[25] Karunai Raj M., Balachandran C., Duraipandiyan V., Agastian P., Ignacimuthu S. (2012). Antimicrobial activity of Ulopterol isolated from Toddalia asiatica (L.) Lam.: a traditional medicinal plant. *Journal of Ethnopharmacology*.

[26] Duraipandiyan V., Ignacimuthu S. (2009). Antibacterial and antifungal activity of Flindersine isolated from the traditional medicinal plant, *Toddalia asiatica* (L.) Lam. *Journal of Ethnopharmacology*.

[27] Hu A. Y., Xie J., Yiao J., Wei Y. (2002). Curative observation of applying LongZhang Gargle to 100 cases of ROU patients. *Journal of Guiyang College of Traditional Chinese Medicine*.

[28] Zhang Y. J., Liu B. Z., Jing F., Peng Y. Z. (2014). Clinical study of Miao medicine gargle on preventing the fixed orthodontic gingivitis. *Journal of Guiyang College of Traditional Chinese Medicine*.

[29] Tian M. L., Chen L. M., Zeng T. Y., Wu H. B. (2001). Clinical study on Longzhang Gargle for root canal disinfection. *Guizhou Medical Journal*.

[30] Lombardo Bedran T. B., Azelmat J., Palomari Spolidorio D., Grenier D. (2013). Fibrinogen-induced *Streptococcus mutans* biofilm formation and adherence to endothelial cells. *BioMed Research International*.

[31] Li M.-Y., Huang R.-J., Zhou X.-D., Gregory R. L. (2013). Role of sortase in Streptococcus mutans under the effect of nicotine. *International Journal of Oral Science*.

[32] Assaf D., Steinberg D., Shemesh M. (2015). Lactose triggers biofilm formation by *Streptococcus mutans*. *International Dairy Journal*.

[33] Bitoun J. P., Liao S., Xie G. G., Beatty W. L., Wen Z. T. (2014). Deficiency of BrpB causes major defects in cell division, stress responses and biofilm formation by *Streptococcus mutans*. *Microbiology*.

[34] Jongsma M. A., van der Mei H. C., Atema-Smit J., Busscher H. J., Ren Y. J. (2015). In vivo biofilm formation on stainless steel bonded retainers during different oral health-care regimens. *International Journal of Oral Science*.

[35] Xiao J., Koo H. (2010). Structural organization and dynamics of exopolysaccharide matrix and microcolonies formation by *Streptococcus mutans* in biofilms. *Journal of Applied Microbiology*.

[36] Qiu W., Zheng X., Wei Y. (2015). D-alanine metabolism is essential for growth and biofilm formation of *Streptococcus mutans*. *Molecular Oral Microbiology*.

[37] Li J. W.-H., Vederas J. C. (2009). Drug discovery and natural products: end of an era or an endless frontier?. *Science*.

[38] Costerton W., Veeh R., Shirtliff M., Pasmore M., Post C., Ehrlich G. (2003). The application of biofilm science to the study and control of chronic bacterial infections. *The Journal of Clinical Investigation*.

[39] Takahashi N., Nyvad B. (2011). The role of bacteria in the caries process: ecological perspectives. *Journal of Dental Research*.

[40] Wong R. W. K., Hägg U., Samaranayake L., Yuen M. K. Z., Seneviratne C. J., Kao R. (2010). Antimicrobial activity of Chinese medicine herbs against common bacteria in oral biofilm. A pilot study. *International Journal of Oral & Maxillofacial Surgery*.

[41] Hu C. H., He J., Eckert R. (2011). Development and evaluation of a safe and effective sugar-free herbal lollipop that kills cavity-causing bacteria. *International Journal of Oral Science*.

